# Recovery of Posture Stability at Different Foot Placements in Patients Who Underwent Minimally Invasive Total Hip Arthroplasty: A One-Year Follow-Up Study

**DOI:** 10.1155/2015/463792

**Published:** 2015-10-25

**Authors:** Chun-Ju Chang, Na-Ling Lin, Mel S. Lee, Jen-Suh Chern

**Affiliations:** ^1^Department of Occupational Therapy and Graduate Institute of Clinical Behavioral Science, Chang Gung University, Taoyuan 333, Taiwan; ^2^Department of Biomedical Engineering, National Yang Ming University, Taipei 112, Taiwan; ^3^Department of Orthopaedic Surgery, Kaohsiung Chang Gung Memorial Hospital, Kaohsiung 833, Taiwan; ^4^Department of Medicine, Chang Gung University, Taoyuan 333, Taiwan

## Abstract

To understand the progression of recovery in postural stability and physical functioning after patients received the minimally invasive total hip arthroplasty (MTHA), we monitor the pain level, functional capacity, and postural stability before and after operation within one year. In total of 23 subjects in our study, we found out that MTHA was effective in relieving pain in first 2 weeks and restoring the hip joint integrity, but the postural stability was influenced especially in tandem stand in both anterior-posterior and medial-lateral directions. The recovery of postural stability and functional capacity in one year duration fluctuated and no consistent improvement tendency was found. We suggested clinicians designing postsurgery rehabilitation program for consistent and progressive long-term recovery of postural stability and fall prevention to optimize surgical results and prevent undesired postoperative consequences.

## 1. Introduction

Hip joint is one of the two most mobile joints in the human body. Due to its location in the body and upright dominated human posture, hip joint not only has to fulfill mobility demands but also has to be able to bear multidirectional loads constantly. Therefore, it is one of the most vulnerable joints developing degenerative osteoarthritis (OA) [1]. The destruction of hip joint due to OA is irreversible and total hip arthroplasty (THA) has been developed several decades ago to reconstruct the joint structure [2, 3]. Advancement of surgical technique to minimize physiological impact during surgical process has been the focus for the recent decade. Minimally invasive THA (MTHA) was, thus, invented and gradually replaces traditional THA [4].

Studies have shown that both THAs are effective in reducing joint pain and MTHA was superior to traditional THA in terms of decreasing blood loss during surgery, decreasing length of hospital stay, fastening walking speed, and daily functional independence [2–4]. However, cross-sectional studies reported subnormal hip joint function even at 24 months after MTHA. However, the impact of MTHA on relieving joint pain, improving functional capacity, and improving postural stability across a one-year period has not been studied [4–6]. It is reasonable to hypothesize that insufficient recovery and lacking of appropriate postsurgery intervention [6, 7] might contribute to the reported incidence of dislocation of the newly replaced join, development of ipsilateral hip OA, and falls after MTHA. Understanding of the progression of recovery of functional capacity and postural stability after MTHA is critical in lengthening the durability of the prostheses, preventing ipsilateral hip joint pathology, and increasing daily living safety [3].

Frequently used outcome measure for MTHA includes self-report visual analogue pain scale (VAS), functional reach tests, Berg Balance Scale, Activities-Specific Balance Confidence Scale [8–11], and self-administered hip-rating questionnaire. Postural stability is unique outcome measures and was used often to quantify the risk of fall in daily living context [12].

Quiet stance is an easy and safe task to perform, compared to level walking for patients with acute and severe unilateral neuromuscular and/or musculoskeletal impairment in lower extremity. Postural stability during quiet stance has been well investigated and is recognized as a dynamic motor control process involving active sensory processing with a constant mapping of perception to action [13]. Somatosensory apparatus located in the neck, trunk, and lower extremities is inevitable in sensing stance perturbation information through upward neural pathway, while trunk and lower extremity muscles are modulated by downward neural pathway formulating matched muscular synergy as postural actions to control stance posture [14]. Efficient postural stability maintains the vertical projection of the center of mass (COM), which is center of gravity (COG), within the configuration of base of support (BOS) to prevent falls [15, 16].

Stance postural stability is frequently quantified by amount of postural sway and patterns of weight distribution over two lower limbs (symmetry versus asymmetry) [13]. The parameters derived from postural sway and weight distribution are the results of ankle and hip movement strategies which actuated through bilateral lower limb loading and unloading mechanism (LULM). It is well accepted that increment of postural sway and asymmetry weight distribution reflect LULM impairments, which in turn indicated insufficient somatosensory and neuromuscular functioning in the lower extremity [13, 15, 16]. However, Chern et al. had suggested that compensatory weight bearing strategy might be developed along with the somatosensory and neuromuscular recovery process to fulfill the priority of daily living safety in patients with unilateral lower limb pathology, such as hemiplegia after stroke, and this compensatory strategy might decrease postural instability [17].

Stance with different foot placements is not uncommon in our daily situation. Foot placement configures the dimension of BOS in both anterior-posterior (AP) and medial-lateral (ML) planes [12]. Changing foot placement perturbs stability through redistributing body mass and deviating COG locations in the dimensions. Therefore, quiet stance postural control features in different foot placement are suitable for and valid in documenting the efficiency of somatosensory and neuromuscular functions of the lower extremity [17], like tandem stand or foot together stand.

With a little knowledge of the progression of functional recovery after receiving the MTHA, it is necessary to monitor the pain level, activity of daily life, and posture stability after the surgery for prevention of falls in daily context and decreasing the need of medical care following falls. The purpose of this study is to investigate pain level, functional capacity, and postural stability in patients with MTHA at pre- and postoperative stages. The postural stability was measured while the subjects stood with four different foot placement conditions. Our hypothesis is that the patients' level of pain, functional capacity, and postural stability would recover progressively at the time immediately after surgery through one year after surgery. The second hypothesis was that the subjects' postural stability would vary as a function of the foot placements which post graded demands on control of postural stability. Therefore, multiple functional and balance measurements were taken before and after the surgery. The results of the study would advise clinicians in designing presurgery education and postsurgery intervention programs for this group of patients to optimize surgical results and prevent undesired postoperative consequences, such as falls.

## 2. Materials and Methods

### 2.1. Participants

Convenient and intentional sampling method was used to recruit participants in this study. A total of 23 participants (10 male and 13 female, mean age of 60 ± 9.9 years, mean height of 165.6 ± 9.7 cm, mean weight of 58.2 ± 11.9 kg, and mean BMI of 23.55 ± 3.50 kg/m^2^) agreed to participate in this study. The inclusion criteria were (1) being diagnosed as unilateral hip arthritis, (2) planning to accept total hip arthroplasty (THA) procedure, (3) being able to stand independently for 3 minutes before surgery, and (4) being able to follow verbal instructions. Comorbidities of the lower extremity, such as osteoarthritis or misalignment at other joints, and other systematic diseases, which might affect stance postural control capability, were the exclusion criteria. A sample size of 23 was justified by statistical power of 0.96 calculated based on an effect size of 0.5. The experimental procedure was approved by Chang Gung Medical Foundation Institutional Review Board (number 97-1389B) and all participants signed informed consent form prior to entering into this study.

### 2.2. Total Hip Arthroplasty

All THAs were performed by an experienced orthopaedic surgeon (the third author) at a medical center, Department of Orthopedic Surgery. All the THAs are minimally invasive but with slight variation in the number and location of incisions. The surgeon chose one of the three procedures based on his professional judgments and patient's choice of hip joint prostheses: (1) modified Watson-Jones minimally invasive approach (MIS-WJ), (2) minimally invasive anterolateral approach procedure (MIS-AL), and (3) two-incision minimally invasive procedure (MIS-2). Incisions were made at either anterior, anterolateral, or posterior aspect of the hip joint. MIS-WJ requires exposure of the hip joint through the muscular intervals [18, 19]; MIS AL involves dissecting of 20–25% of gluteus medius between anterior border of gluteus medius and tensor fasciae latae. All procedures involved dissection across and blunt dissection of the gluteus maximus to create space for orthotic stem implantation [20–22]. All the incisions were within 10 centimeters long with the shortest incision of 2–6 cm in MIS-2 approach [23–26].

### 2.3. Experiment Procedures and Instrumentations

After signed informed consent form, participants were arranged for measurement of pain level, functional capacity of the hip joint, and postural stability as the dependent variables. All measurements took place at 1 day before surgery and 2 weeks, 6 weeks, 3 months, 6 months, and 1 year after surgery. The time point and the foot placement described as follows would be the independent variables in our study. Pain level was measured using visual analogue pain scale (VAS, 1: no pain at all, 10: extreme pain). Functional capacity was measured by functional reach tests (FRT), Berg Balance Scale (BBS), self-administered hip-rating questionnaire (hip score), functional independence measure (FIM), and Activities-Specific Balance Confidence Scale (ABC). FRT is a quick screen tool for risk of falls in adults with or without balance deficits. The individual is asked to firstly stand erect with their feet shoulder width apart, secondly elevate one arm at 90° shoulder flexion with hand closed as a fist, and finally slide a yard (which is fixed on the wall at acromion height) as far as possible without moving their feet or losing balance [27]. BBS is a 14-item test with a 4-point rating scale. It is a performance-based test with standardized rating criteria. The total score ranges from 0 to 56 [12]. Hip score is both a self-report questionnaire and subjective measurement tool. It is administered routinely in orthopaedic clinics to monitor progression of hip function for patients with hip problems. The total score ranges from 0 to 100. A score less than 70 indicates poor hip capacity and a score over 90 indicates excellent hip function [28]. FIM is a measure of level of disability in daily living context with a 7-point rating scale. The total score ranges from 18 to 126, and it was used to justify the need of assistance of patients [29]. The ABC is a 16-item test with an 11-point scale and ratings consist of whole numbers (0–100) for each item. The possible range of the item is 0–1600. The total rating is divided by 16 to get each subject's ABC score. ABC score less than 67 indicates a risk of falls and a score more than 80 indicates high level of physical functioning [10]. The psychometric characteristics of all functional tests used in this study were established and testing and rating process were standardized.

To measure postural stability, participants were instructed to stand barefoot with 4 foot placements as shoulder width stance (SWS), feet side-by-side stance (SSS), tandem stance with affected limb in the front (AFS), and tandem stance with nonaffected limb in the front (SFS) on a 0.5 m long pressure measurement mat (RSscan International Co., Belgium) and were instructed to stand as still as possible with both arms at their sides and eyes staring at a target 5 m away in front of them. The pressure mat is 488 mm × 325 mm containing 4096 sensors and the maximum sampling rate is 500 Hz. In each foot placement condition, three trials lasting 30 s (sample frequency 30 Hz) were recorded with 15 s resting interval between trials. To eliminate the unstable data before an individual stands still, the instrument started to collect data at 5 s after the subject stood on the mat. To avoid the order effects, the sequence of four foot placements was randomized.

All patients administered for MTHA received limited postsurgery rehabilitation during either their hospital stay (which is 3 to 5 days) for surgical procedure or outpatient follow-up after surgery because the financial reimbursement of the National Health Insurance System is very limited. Bedside education before and after surgery regarding the hip precautions, such as prohibited motion type and range [30] after MTHA, is given by either nurses or physical therapists in a short counseling session. No regular rehabilitation program was prescribed by surgeons and rehabilitation specialists were not regular members of the team providing medical care for MTHA patients. There is no active training effort initiated or imported by any individual in the team. No more regular rehabilitation was provided after the surgery and the progression of functional recovery in those patients was not monitored.

### 2.4. Data Processing

Average value and standard deviation of all measures were used for statistical analysis. Stance postural control actions were measured by a pressure measurement mat which was connected with a 3D data processing unit and 2-dimensional coordinates of center of pressure (CoP) in anterior-posterior (AP) and medial-lateral (ML) axis and the amount of weight loaded under each limb was output to a customized written program for calculation of parameters. The parameters frequently used for representing stance postural stability include resultant CoP sway path length (CoPR), maximum CoP sway in ML direction (CoPML), maximum CoP sway in AP direction (CoPAP), and body weight distribution symmetry index (BWDSI). The amplitude of CoP parameters is determined by the movement strategy which is actuated by LULM. Normalization of CoP parameters is not necessary because of repeated measure design in this study. Calculation of BWSDI is shown in formula (1). The closer the BWDSI is to the value of zero, the more symmetrical the bilateral weight distribution over two lower limbs is, indicating that both lower limbs are able to bear body weight equally:

(1) $$\begin{array}{c} \text{BWDSI}=\frac{\left({\text{Load}}_{\text{UO}}-{\text{Load}}_{\text{O}}\right)}{\left({\text{Load}}_{\text{UO}}+{\text{Load}}_{\text{O}}\right)}*100. \end{array}$$

Load_UO_ is body weight loaded over unoperated limb. Load_O_ is body weight loaded under operated limb.

### 2.5. Statistical Analysis

Firstly, the function of boxplot in the MATLAB 2012a (Mathworks, Natick, MA) was used for excluding the outlier values of measured variable. The Shapiro-Wilk test for small sample size was used to confirm the normal distribution. And then, the effects of time points on pain level and functional capacity were examined by one-way repeated measure ANOVA. Interaction effects of foot placements and time points on measures of postural stability were examined by two-way repeated measure ANOVA, and degree of freedom was adjusted based on Mauchly's test of sphericity assumption. When the interaction effects were significant, simple main effects of both time points and foot placement conditions were examined by one-way repeated measure ANOVA. Finally, Tukey's HSD test was used as post hoc analysis to show the difference between time points or the foot placements. Statistical significance was set at the level of *p* < 0.05. Commercialized statistical software SPSS 20.0 for windows package (IBM Corp., Armonk, NY) was used for all statistical analysis.

## 3. Results

### 3.1. Primary Outcomes

There was significant difference in BBS, FIM, VAS, and hip scores among time points (Table 1, *p* < 0.01). Subsequent analysis showed that level of pain decreased significantly (*p* < 0.05) at 2 weeks postoperatively but increased slightly starting at 6 months postoperatively. The hip function as measured by hip score tended to improve gradually and the highest score of 85.0 ± 11.6 was reached at 1 year after surgery. The daily living function as measured by FIM decreased significantly (*p* < 0.05) at 2 weeks and reached plateau score of 125.8 ± 0.6 at 6 months postoperatively. Berg balance test decreased significantly (*p* < 0.05) at 2 weeks and improved gradually, reaching the highest score of 53.2 ± 1.9 at 6 months. The difference in functional reach test in all three directions was approaching significant level (Table 1, *p* = 0.07–0.08). The descriptive data showed that functional reach distance tended to improve gradually after surgery, reached the maximum distance, and decreased at 1 year postoperatively.

### 3.2. Effects of Foot Placement on Stance Postural Stability

**Figure 1:**
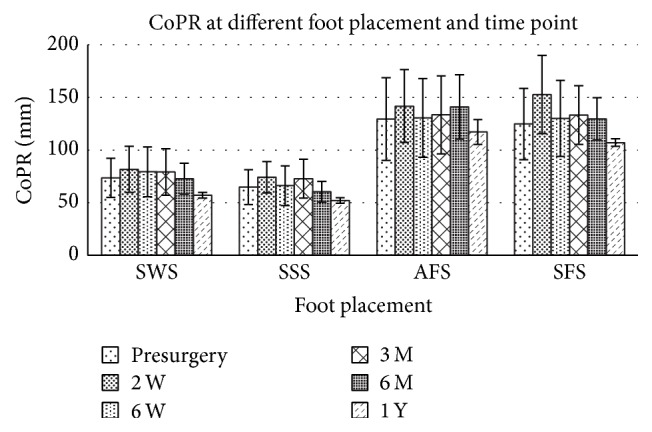
CoP sway path length (CoPR) at different foot placement and time points before and after surgery. Shoulder width stance (SWS), feet side-by-side stance (SSS), tandem stance with affected limb in the front (AFS), and tandem stance with nonaffected limb in the front (SFS).

**Figure 2:**
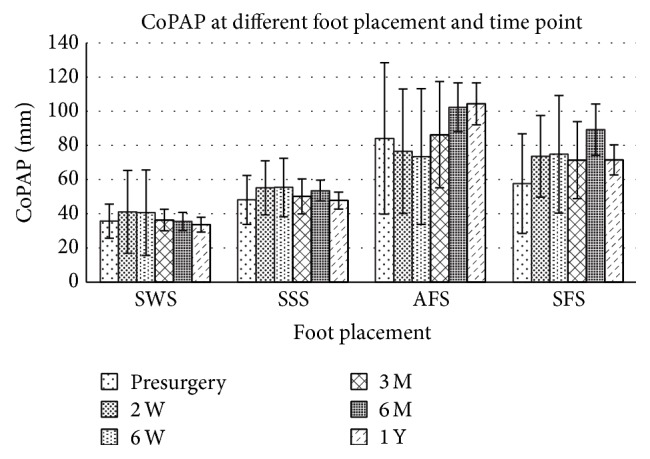
Maximum CoP sway in AP direction (CoPAP) at different foot placement and time points before and after surgery. For abbreviation of foot placement, refer to Figure 1.

**Figure 3:**
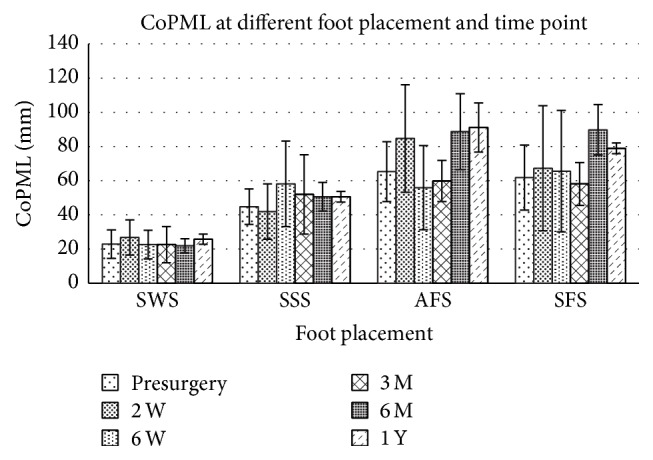
Maximum CoP sway in ML direction (CoPML) at different foot placement and time points before and after surgery. For abbreviation of foot placement, refer to Figure 1.

**Figure 4:**
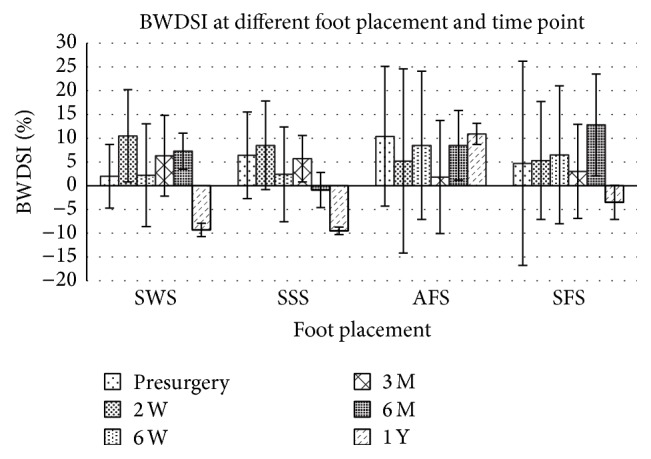
Body weight distribution symmetry index (BWDSI) at different foot placement and time points before and after surgery. For abbreviation of foot placement, refer to Figure 1.

The interaction effects between foot placement and time point relative to surgery date were significant in all measures (Table 2, *p* = 0.000–0.018), indicating that the effects of foot placement on standing stability depended on the time point when the sway was measured. Subsequent main effect analysis found that foot placement affects CoPR, CoPAP, and CoPML significantly (Table 2, main effect of foot placement factor *p* < 0.01) at both presurgery and postsurgery time points. When the foot placement changes from shoulder width stance (SWS), side-by-side stance (SSS), and nonaffected limb in the front stance (SFS) to affected limb in the front stance (AFS), the subjects tended to increase their postural sway after surgery and the progressive increment of CoPR (Figures 1–4). Descriptive data further showed that the amplitude of CoPR is the least when standing in the position of SSS and the greatest during AFS and SFS position (Table 2). The CoP sway in both the CoPAL and CoPML direction was affected significantly by foot placement especially when the width of BOS was narrowed down significantly by foot placement in tandem stance (Figures 2 and 3).

The average bilateral weight bearing symmetrical index was not affected by foot placement (Table 2, *p* = 0.129) but by time point (Table 2, *p* < 0.01). Before and after surgery, the THA patients tended to load their nonaffected limb slightly more than the affected limb when the subjects stood in SWS, SSS, and SFS posture until 1 year after the surgery. When they stood in AFS posture, the subjects tended to increase the weight loaded over the affected limb (Figure 4). At 6 months after surgery, the subjects started to load their weight over the affected limb more than the nonaffected limb only when they adopted SSS posture (Table 2). At 1 year after surgery, the subjects loaded their weight over the affected limb more than the nonaffected limb with almost all four stance postures except when they stood with the affected foot in the back tandem stance (AFS).

### 3.3. Effects of Time Points on Stance Postural Control Features

The effects of time points on stance postural stability depended on which stance posture the subject adopted. The CoPR, CoPML, and BWDSI showed different across time point in all four foot placements (Table 2), but CoPAP had no main effect on the factor of time point (*p* = 0.185). Subsequent analysis showed that the effects of time point on CoPR were not consistent across foot placement. The CoPR had the greatest postural sway at the time of 2 weeks after surgery, but the CoPML at the time of 6 months after surgery. The descriptive data showed that the CoPR tended to decrease progressively after surgery (Figure 1). CoPAP peaked between 6 months and 1 year after surgery (Figure 2), especially at the AFS and SFS position.

## 4. Discussion

This is the first and the only study investigating the pain level, functional capacity, and postural stability in patient who underwent MTHA with 1 year follow-up. Quiet stance postural stability during four foot placement conditions was measured to represent the ability of hip joint to manage graded demands for control of postural stability. Though quiet stance has been called a “static” postural control task because the base of support (BOS) is not changing, several studies [31, 32] have suggested that quiet stance is dynamic sensorimotor tasks requiring a very precise control of fine movements of the lower extremities by unceasingly displacing the location of CoP and modulating interlimb loading ratio. Studies also showed that both musculoskeletal and neuromuscular impairments can change the amount of CoP movement and the ratio of interlimb weight bearing [13]. Therefore, postural control actions while quiet stance was measured in this study at six time points before and after MTHA to reveal their sensorimotor recovery process around hip joint. In general, our results showed that MTHA in this study did relieve hip pain and improve function capacity effectively (Table 1). The sensorimotor function around the hip during control of postural stability tended to recover irregularly, and the hypotheses in this study were therefore partially supported.

Our results first showed that the functional capacity of the hip was affected by MTHA but the trend of progressive improvement was not observed through 1 year after surgery. As previously reported [33], the most prominent effects of MTHA were on decreasing the hip pain and on increasing independence of daily functions. However, the MTHA tended to affect postural stability positively within 6 months but negatively within 6 months after surgery as shown by measures of Berg Balance Scale [34, 35] and functional reach test [17], indicating that the somatosensory and neuromuscular function around hip joint did not recover progressively or completely within 1 year. The results of muscle strength further showed that a single joint surgery, even with minimally invasive approach, affects not only the hip muscle strength but also muscle strength around the remote joint in the lower extremity, indicating that the muscular synergy for control of stance postural stability might be also affected [1, 36]. Moreover, our results showed that the stance postural stability during different foot placement conditions was influenced by time points at which postural stability was measured, indicating that the performance of neuromuscular apparatus around hip joint in the process of stance postural control depends on both the configuration of BOS and level of recovery after surgery [13, 31, 32]. The following discussion was based on simple main effects of foot placement and time points on stance postural control features to elucidate the impact of MTHA on hip joint sensorimotor functions.

### 4.1. Simple Main Effects of Time Points on Stance Postural Control Features

Our results showed significant alteration of postural actions between pre- and postoperative stages and between 6 months and 1 year after surgery. The measured postural stability seemed to alter in accordance with subjective feeling of pain or discomfort [2, 3]. As shown in Tables 1 and 2 the joint pain was relieved significantly and immediately after surgery. However, the patients started to report feelings of discomfort and joint tightness at six months or one year after surgery. This result suggested that level of pain associated with hip joint structural integrity but not with hip joint functional integrity. The progression of stance stability was consistent with Berg balance score and functional reach tests. The results of FIM and hip scores [37–39] suggested that the patients with MTHA tended to be more confident than they are before surgery in daily functioning. The good side of these results is that the autonomy of the patients increased which might accompany increase of quality of life. On the other hand, the patient might put themselves in danger of falling because deficits in stance stability (as shown in Table 2) might emerge and/or last at least until one year after surgery. Clinicians and patients should both be advised and the patients should be instructed to follow postsurgery precautions even though the pain decreased significantly within three months after surgery [2, 3].

Interestingly, the variability of postural stability, as shown by standard deviation measured, was quite large at each time point postoperatively, although the average postural actions remained constant across time point between 2 weeks and 6 months after surgery. The explanation of the large variability might be due to inconsistent postsurgery rehabilitation regimen. Several studies have shown that after 4 weeks of postoperative rehabilitation programs, THA patients were able to achieve an almost complete restoration of the lower limb function and hip joint range of motion and independence in the activities of daily living [8, 40]. In general, the rehabilitation program was implemented by health professionals beginning on the first postoperative day and continued in rehabilitation unit on inpatient basis at 5 days postoperatively [32, 41]. The main goal of rehabilitation was to improve range of motion, muscle strength, aerobic capacity and activities of daily living, and functional abilities such as standing and walking. However, the patients in our study did not have access to standard rehabilitation resources. They are usually educated pre- and postoperatively regarding the wound care and movement precautions after surgery during their short hospital stay (usually 3 to 5 days). The inpatient and postsurgery rehabilitation includes teaching of use of a walker and/or crutches for indoor ambulation and isometric exercises for the operated limb [36]. Finally, the surgeon usually advises the patients to move around as much as possible after they were discharged to home at 3 to 5 days after surgery. Our data with fluctuated postural stability after surgery indicated that the patients might not train themselves enough and self-training might vary across each individual. Clinical interview of our patients showed that some of them tended to decrease their activity level after THA because they were afraid of dislocation of the newly operated joint or OA over the nonoperated joint; others would participate in aerobic activity such as swimming to facilitate functional ability. No therapist was involved in the rehabilitation treatment; either specific ROM or muscle strength training instruction was provided. This might cause the feeling of joint tightness or discomfort that developed at six months after surgery and, thus, change the postural stability while standing. Whether activity levels and self-training intensity facilitate neuromuscular outcomes in patients who underwent MTHA should be further investigated [42–44].

Another noteworthy factor contributing to the fluctuated stance postural stability might be different incision approaches used by the same surgeon across patients. Although the THAs in this study fulfilled the requirement of minimally invasive technique, the level of soft tissue damaged and the muscle groups injured varied across approaches [45–48]. Our results suggested that the neuromuscular outcomes among four MTHAs might be different. Previous studies reported on asymmetric limb kinetic performances during walking in patients who underwent two approaches of MTHA [9, 49]. Further studies should compare the outcomes among three different approaches used.

### 4.2. Simple Main Effects of Foot Placements on Stance Postural Control Features

Our results have highlighted the tendency of increased postural sway as the foot placement changes from bilateral feet shoulder width stance, bilateral feet side-by-side stance to tandem stance, as reported by previous studies investigating upright standing control features in stroke or normal subjects [17, 50]. These results indicated that the upward neural pathway responsible for perceiving changes in dimensions of BOS in patients with MTHA might be active and efficient for modulation of postural actions, as reported by other studies. On the other hand, the significant increment of postural sway during tandem stance comparing with other stance postures might indicate that the MTHA induced limited neuromuscular capacity around hip joint [9] to constrain postural sway when the demands of postural control increased due to change of BOS dimension. Therefore, it is reasonable to hypothesize that the risks of fall in patients who underwent MTHA might be higher when they stood with tandem stance posture than with the other two stance postures. We, therefore, suggest that pre- and postoperative education programs, in addition to prohibiting certain hip joint movements, should advise the patients to avoid adopting tandem stance posture for at least three months to prevent falls [51].

Another striking result is that patients with MTHA tended to load the newly operated hip more than the nonoperated limb during tandem stance without cautions (Figure 3). Inappropriate weight loading over the prosthetic limb was reported to not only increase the incidence of dislocations but also decrease the durability of the prostheses. Therefore, clinicians should advise the patients to avoid standing with heel-toe posture to decrease the incidence of dislocation over the newly operated hip caused by inappropriate weight acceptance [41, 51]. On the other hand, the asymmetric limb loading pattern shed light on the significant contribution of impairment of interlimb LULM on increment of CoP sway magnitude increment. Significant decrement of pain over the OA hip makes the patients subconsciously load the operated limb intermittently but the insufficient hip muscular strength, as proposed by several previous studies [17, 52–54], might cause the frequent interlimb loading and unloading. The weighting of painful sensation and muscular capacity on changes of limb loading patterns and CoP sway magnitude should be further investigated.

Another noteworthy result in this study is that the influence of foot placements on CoP sway in AP and ML direction is different and this phenomenon is the biomechanical consequence of alteration of the dimension of BOS in AP and ML aspects [13]. Shoulder width stance (SWS) and bilateral feet side-by-side stance (FSS) configure the same dimension of BOS in AP aspect but the dimension of BOS in ML aspect is wider in SWS than in SFS. Both tandem stances (AFS and SFS) configure the same dimension of BOS in ML aspect, but the dimension of BOS in AP aspect in SFS is much longer than in SWS and in AFS. Our results found that the CoPAP tended to increase not only as the BOS lengthened in AP dimensions but also as the BOS remained constant in AP aspect. As reported by Rougier [31], the CoP sway in AP aspect is mainly modulated by ankle joint motion and was weakly linked with bilateral limb loading pattern which is primarily modulated by hip joint motion. Therefore, our finding, as those reported [25], suggested that though MTHA affects only the hip joint, it could cause impairments of interjoint coordination (between hip, knee, and ankle joints) necessary for bipedal stance postural control. In ML aspect, our data indicated that CoPML linked with asymmetric limb loading pattern and the COPML was affected by alteration of BOS dimension in ML aspect more than COPAP was. This result is consistent with Rougier's finding [31] and is not surprising since CoP sway in ML aspect is mainly achieved by the control of hip abductor and adductors [42], indicating that MTHA impaired significantly the sensorimotor function and thus limits patient's capacity to effectively constrain postural sway in ML aspects according to the dimension of BOS.

The associated asymmetrical limb loading further indicated that the ankle mechanism of the patients with MTHA is not able to compensate the impaired hip mechanism and, therefore, an adaptive strategy to constrain CoP sway after surgery was not seen. Further studies analyzing relationship between reaction forces, COP sway under each foot, and muscular activation pattern are necessary to understand the adaptive strategies at different foot placement in patients with MTHA.

The major limitation of this study was that the researchers responsible for data collection were not blinded to the purpose of this study. This might impede the objectivity of the data collected when testing the functional capacity. The reason that had caused this limitation is the limited personnel resource allocated. The second limitation was that MTHA with four different approaches was used. Different approaches used might cause the prominent variability of postural stability observed in this study. Small sample size is another limitation which is not uncommon among clinical studies.

## 5. Conclusions

MTHA is a frequently used procedure to alleviate functional deficits caused by hip joint osteoarthritis. This study found that MTHA is effective in relieving pain in first 2 weeks and restoring the hip joint integrity. However, deterioration of joint pain and functional capacity starting at 6 months postoperatively was noted and these results were associated with sensorimotor recovery fluctuation. Lack of standard and regular postoperative rehabilitation might contribute to this fluctuation. The results of this study also indicated that stance postural stability in patients who underwent MTHA was influenced by foot placement and inappropriate postural stability in both AP and ML directions was noted, especially when the patients stood in tandem posture. Advisement of establishing standardized and regular postsurgical rehabilitation program, including advising the patients to avoid standing postures which expose the operative joint and/or the ipsilateral limb to danger, is recommended for preventions of falls, prolonging the usable duration of the prosthesis and reducing the needs of medical care. Effects of postsurgical rehabilitation program need to be clarified with more delicate research design (such as blindness of the researchers responsible for data collection and inclusion of larger sample size). Comparison among four different MTHA approaches for their effects on functional capacity and sensorimotor recovery is inevitable for clinical decision making.

## Figures and Tables

**Table 1:** One-way repeated measure ANOVA summary showing the effects of time points on functional assessment.

Functional assessment	Time points before and after surgery						*p* ^†^
	Presurgery	2 w	6 w	3 M	6 M	1 yr	
Berg balance test (56)	44.6 ± 7.3^ac^	36.4 ± 12.9^b^	41.4 ± 7.9^bc^	47.7 ± 4.7^ac^	53.2 ± 1.9^a^	52.5 ± 3.3^a^	<0.0001^*∗∗*^
Functional reach test (cm)							
Forward	22.2 ± 6.7	22.6 ± 4.2	24.6 ± 5.3	20.4 ± 5.1	28.7 ± 7.6	27.4 ± 7.5	0.0854
Toward affected side	19.8 ± 4.1	20.9 ± 9.4	19.4 ± 3.6	18.9 ± 3.6	25.7 ± 4.7	22.2 ± 5.2	0.0828
Toward unaffected side	18.8 ± 5.2	18.5 ± 3.2	18.6 ± 4.4	18.3 ± 8.7	25.3 ± 2.8	23.7 ± 4.5	0.0712
ADL independence/pain							
VAS^*∗*^ (10)	4.8 ± 1.4^a^	2.3 ± 1.6^b^	1.1 ± 1.2^b^	1.1 ± 1.0^b^	1.4 ± 1.0^b^	2.1 ± 2.3^b^	<0.0001^*∗∗*^
Hip score^*∗*^ (100)	63.2 ± 9.3^a^	65.0 ± 9.8^a^	74.6 ± 9.8^b^	83.6 ± 10.3^bc^	82.7 ± 9.5^b^	85.0 ± 11.6^c^	<0.0001^*∗∗*^
FIM^*∗*^ (126)	119.7 ± 4.6^a^	110.2 ± 19.6^b^	118.0 ± 5.8^ab^	122.3 ± 4.0^a^	125.7 ± 0.7^a^	125.8 ± 0.6^a^	<0.0001^*∗∗*^

^*∗*^Visual analogue pain scale (VAS); self-administered hip-rating questionnaire (hip score); functional independence measure (FIM).

^†^One-way repeated measure ANOVA. Tukey's HSD test for comparison of the period and the same letter means no significant difference.

^*∗∗*^ *p* < 0.01.

**Table 2:** Summary of two-way repeated measure ANOVA examining the interaction of time point and foot placement and subsequent post hoc analysis of parameters representing postural stability.

Time points	Foot placement						
	SWS	SSS	AFS	SFS	*p* ^i^	*p* ^t^	*p* ^f^
CoPR (unit: mm)							
Presurgery	73.5 ± 18.6^a1^	64.7 ± 16.6^a1^	129.3 ± 39.2^a2^	124.7 ± 33.8^a2^	0.003^*∗∗*^	0.000^*∗∗*^	0.000^*∗∗*^
Postsurgery, 2 wk	81.8 ± 21.9^a1^	74.1 ± 14.8^b1^	141.8 ± 34.6^ab2^	152.7 ± 37.1^b2^			
Postsurgery, 6 wk	79.3 ± 23.7^a1^	66.1 ± 18.8^a1^	130.6 ± 37.4^ad2^	130.1 ± 36.0^a2^			
Postsurgery, 3 mo	79.2 ± 22.1^a1^	72.8 ± 18.5^a1^	133.4 ± 37.1^a2^	133.2 ± 27.8^a2^			
Postsurgery, 6 mo	72.8 ± 14.5^ab1^	60.5 ± 9.9^ac1^	141.0 ± 30.5^a2^	129.6 ± 20.0^ac2^			
Postsurgery, 1 yr	57.0 ± 2.7^b1^	52.1 ± 2.6^c2^	117.2 ± 11.7^acd3^	107.1 ± 3.5^acd4^			
CoPAP (unit: mm)							
Presurgery	35.7 ± 10.0^a^	48.1 ± 14.3	84.1 ± 44.3^a^	57.6 ± 29.1^a^	0.018^*∗*^	0.185	0.000^*∗∗*^
Postsurgery, 2 wk	41.1 ± 24.3^b^	55.1 ± 15.8	76.6 ± 36.4^a^	73.6 ± 23.9^b^			
Postsurgery, 6 wk	40.6 ± 25.0^bc^	55.4 ± 17.1	73.5 ± 39.7^a^	74.8 ± 34.5^bc^			
Postsurgery, 3 mo	36.3 ± 6.3^ab^	50.1 ± 10.2	86.2 ± 31.1^a^	71.3 ± 22.5^bd^			
Postsurgery, 6 mo	35.5 ± 5.3^ab^	53.5 ± 6.1	102.3 ± 14.3^ab^	89.2 ± 15.0^be^			
Postsurgery, 1 yr	33.6 ± 4.3^ab^	47.7 ± 5.0	104.3 ± 12.3^b^	71.5 ± 8.8^ab^			
CoPML (unit: mm)							
Presurgery	22.9 ± 8.3^a1^	44.8 ± 10.4^2^	65.3 ± 17.5^a3^	61.8 ± 19.0^a34^	0.003^*∗∗*^	0.000^*∗∗*^	0.005^*∗∗*^
Postsurgery, 2 wk	26.8 ± 10.3^ac1^	41.9 ± 16.2^2^	84.7 ± 31.5^ac3^	67.3 ± 36.6^ab34^			
Postsurgery, 6 wk	22.7 ± 8.4^a1^	58.2 ± 25.0^2^	55.9 ± 24.6^a3^	65.5 ± 35.3^ab34^			
Postsurgery, 3 mo	22.6 ± 10.5^ab1^	52.0 ± 23.2^2^	59.8 ± 12.0^ad3^	58.1 ± 12.5^a34^			
Postsurgery, 6 mo	21.9 ± 4.1^ab1^	50.6 ± 8.4^2^	88.7 ± 22.1^b3^	89.8 ± 14.8^bc34^			
Postsurgery, 1 yr	25.8 ± 3.0^a1^	50.6 ± 3.1^2^	91.1 ± 14.4^b3^	78.9 ± 3.2^ac34^			
BWDSI (unit: % of body weight)							
Presurgery	2.0 ± 6.7	6.4 ± 9.1	10.4 ± 14.7	4.7 ± 21.5	0.000^*∗∗*^	0.000^*∗∗*^	0.129
Postsurgery, 2 wk	10.5 ± 9.7^1^	8.5 ± 9.3^13^	5.2 ± 19.4^2^	5.3 ± 12.4^23^			
Postsurgery, 6 wk	2.2 ± 10.8	2.4 ± 10.0	8.5 ± 15.6	6.5 ± 14.5			
Postsurgery, 3 mo	6.3 ± 8.5^1^	5.7 ± 4.9^1^	1.8 ± 11.9^23^	3.0 ± 9.9^13^			
Postsurgery, 6 mo	7.3 ± 3.8^1^	−0.9 ± 3.7^2^	8.5 ± 7.3^1^	12.8 ± 10.7^1^			
Postsurgery, 1 yr	−9.3 ± 1.4^1^	−9.5 ± 0.8^1^	10.9 ± 2.2^2^	−3.5 ± 3.6^3^			

Shoulder width stance (SWS), feet side-by-side stance (SSS), tandem stance with affected limb in the front (AFS), and tandem stance with nonaffected limb in the front (SFS).

*p* ^i^: interaction between factors; *p* ^t^: main effect of time; *p* ^f^: main effect of foot placement; ^*∗∗*^ *p* < 0.01; ^*∗*^ *p* < 0.05.

Superscript of alphabet: Tukey's HSD post hoc analysis of simple main effect of foot placement factor.

Superscript of number: Tukey's HSD post hoc analysis of simple main effect of time factor.
